# Social cues to joint actions: the role of shared goals

**DOI:** 10.3389/fpsyg.2015.01034

**Published:** 2015-07-30

**Authors:** Lucia M. Sacheli, Salvatore M. Aglioti, Matteo Candidi

**Affiliations:** ^1^Department of Psychology, University of Rome “Sapienza”, Rome, Italy; ^2^Istituto di Ricovero e Cura a Carattere Scientifico, Fondazione Santa Lucia, Rome, Italy; ^3^Department of Psychology, University of Milano-Bicocca, Milan, Italy

**Keywords:** joint-action, shared goals, socio-emotional context, interpersonal perception, kinematics, grasping

## Abstract

In daily life, we do not just move independently from how others move. Rather, the way we move conveys information about our cognitive and affective attitudes toward our conspecifics. However, the implicit social substrate of our movements is not easy to capture and isolate given the complexity of human interactive behaviors. In this perspective article we discuss the crucial conditions for exploring the impact of “interpersonal” cognitive/emotional dimensions on the motor behavior of individuals interacting in realistic contexts. We argue that testing interactions requires one to build up naturalistic and yet controlled scenarios where participants reciprocally adapt their movements in order to achieve an overarching “shared goal.” We suggest that a shared goal is what singles out real interactions from situations where two or more individuals contingently but independently act next to each other, and that “interpersonal” socio-emotional dimensions might fail to affect co-agents’ behaviors if real interactions are not at place. We report the results of a novel joint-grasping task suitable for exploring how individual sub-goals (i.e., correctly grasping an object) relate to, and depend from, the representation of “shared goals.”

## Introduction

“The difference between a helping hand and an outstretched palm is a twist of the wrist”

L. Leamer, King of the Night.

In order to explore the neuro-cognitive bases of interpersonal encounters, social neuroscience needs to shift from “isolation paradigms” (Becchio et al., 2010), which investigate “offline” social cognition from the point of view of a (passive) observer (Pfeiffer et al., 2013), to an active, “second-person” approach (Schilbach et al., 2013), which validates the idea that—in real life—“online” social interaction is much more than just the concurrent recruitment of the essentially isolated social knowledge of individuals (see also Gallotti and Frith, 2013). This implies adopting experimental set-ups that (i) explore the emergence of closed-loop processes (i.e., allowing partners’ reciprocal adjustments during the interaction), and (ii) take into account the emotional engagement that characterizes social encounters (Schilbach et al., 2013).

This issue becomes essential when studying “joint actions (JAs),” which we refer to here, defined as activities involving two or more individuals who need to coordinate their actions in time and space with the aim to realize together a desired change in the environment (Sebanz et al., 2006). This scenario requires dynamic experimental paradigms where the agent’s individual goal is inherently linked to that of a partner thus depending on mutual adjustments, and where participants perceive themselves as a “couple” which is acting together as a unity *because* they share an overarching common goal. In the present perspective article we suggest that only such experimental paradigms will allow scholars to study the “socio-emotional nature” of interpersonal coordination and will create a context suitable for exploring whether, and how, socio-emotional variables impact the quality of the interaction. We will focus on on-line interactions that—in our view—best highlight why mutual adjustments are based on shared goals. Indeed, while in turn-taking situations each individual is “passive” at some point during the interaction, on-line interactions require synchronicity in space and time: thus, they require that co-agents actively understand the partner’ behavior and predict his/her action goal while also monitoring their own action execution. This situation requires adapting one’s own action goal to a shared representation of the interaction. This is not a mere difference in complexity, but it is a difference in quality: without the constraint of synchronicity, the interaction may reproduce a condition where one individual is a passive observer.

In what follows, we try and provide an experimentally useful definition of “shared goals,” and we describe why we believe shared goals single out JAs from situations where an agent passively observes or mechanically react to the actions of other individuals. Then, we will operationalize how shared goals can be investigated in a well-controlled interactive task and explain why the analysis of kinematics in general (see, for instance Noy et al., 2011; D’Ausilio et al., 2012; Vesper et al., 2013; Vesper and Richardson, 2014) and grasping kinematics in particular (Georgiou et al., 2007; Becchio et al., 2008a,b; Sartori et al., 2009) might be a powerful instrument to explore the neuro-cognitive instantiations of shared goals. Thus, we will describe a set-up that we specifically designed to investigate the relation between individual and joint goals during an interactive grasping task (Sacheli et al., 2012, 2013, 2015). Our studies provide empirical evidence that motor tasks that include shared goals are suitable for exploring the impact of the socio-emotional context on planned interpersonal coordination.

## Defining Shared Goals

Although there is evidence of “proto” forms of cooperative activities in non-human species (Mendres and de Waal, 2000; Seed et al., 2008; Plotnik et al., 2011), studies suggest that the tendency to interact and pursue common goals is typically human and shows up early in development (Tomasello et al., 2005; Warneken et al., 2006). Importantly, the tendency to share goals and intentions with others might support the establishment of social bonds: the efficacy of the interaction itself and the emotional reactions to it may also influence the process of coding others as in-group or out-group members (Hommel et al., 2009; Iani et al., 2011).

In the present perspective article, we focus on (on-line) JAs as a way to realize shared goals. Influential studies suggest that performing successful joint-actions depends on the ability to: (i) share representations, (ii) predict others’ actions, and (iii) integrate predicted effects of one’s own and others actions (Sebanz et al., 2006). Crucially, this definition highlights that interacting individuals cannot directly access a partner’s motor plan and thus need to infer it from his/her overt behavior (aside from environmental cues). Moreover, since reactive processes do not suffice in supporting the fine-tuned temporal contingency required by on-line interpersonal coordination, co-agents cannot simply react to the partner’s behavior but need to predict it (Knoblich and Jordan, 2003). Predictive coding is (at least partially) based on predictive sensorimotor processes triggered by the observation of others’ actions (Kilner et al., 2007). However, a fundamental question concerns *what* is actually “shared” of motor representations during on-line interpersonal interactions. We suggest that “shared goals” (Butterfill, 2012) create a link between interacting co-agents by integrating, in a unique motor plan, the representation of one’s own and a partner’s action (see also Knoblich et al., 2011).

According to Butterfill (2012), three features of shared goals are crucial: (i) there is a single shared goal, *G*, to which each agent’s actions are (or will be) individually directed; (ii) each agent expects each of the other agents to perform an action directed to the shared goal *G*; (iii) each agent expects this goal *G* to occur as a common effect of all actions directed toward it, i.e., both his or her own and the partner’s ones. Thus, a shared goal is both “in common” between co-agents and “divided up” into individual sub-goals that each actor needs to achieve to fully accomplish the intended JA.

In keeping with computational (Wolpert and Ghahramani, 2000) and neurophysiologic studies (Fogassi et al., 2005; Grafton and Hamilton, 2007) indicating that the motor system represents individual goals according to a hierarchical structure, we suggest that JA and shared goal representations are not independent from this organization: just as individual muscular synergies are coordinated in complex actions by the need to achieve a desired (individual) motor goal, interpersonal motor synergies are shaped by the presence of shared goals which organize co-agents’ behaviors (Chersi, 2011; Candidi et al., 2015). In keeping, framing research on JA as *research on actions involving two or more agents sharing a common goal* implies suggesting JAs are characterized by a “hierarchical structure” where the accomplishment of a (shared) overarching goal depends on the fulfillment of the sub-goals that each interacting partner is required to achieve. For instance, the overarching common goal of moving a table together is achieved only when both partners accomplish their own individual sub-goal (e.g., pulling and pushing the table in the right direction) by dynamically adapting to each other in space and time.

Importantly, the definition of “shared goals” provided above does not overlap with the one of “shared representations” as they have been defined by studies on joint attention (Sebanz et al., 2003, 2005, 2007). Studies on joint attention typically investigate conditions where one binary choice task with two competitive target stimuli is split between two participants, with each participant responding to only one of the targets in turn-taking because he/she has “his/her own target” to respond to (e.g., paradigms leading to joint Simon effect, Tsai et al., 2008; Flanker effect, Atmaca et al., 2011, and SNARC effect, Atmaca et al., 2008). In these tasks, participants have to attend to one target and to ignore the other. Thus, in principle their performance in the joint condition should resemble the one in individual go/no-go tasks. However, participants involuntarily take the co-actor’s task into account albeit they are explicitly instructed to ignore it. This suggests that humans have a tendency to form “task co-representations” which specify not only one’s own but also a co-actor’s task, even if the co-actor’s task is irrelevant to (or even interfering with) one’s own task fulfillment. Although the ability to co-represent a task is obviously crucial in JA, the above studies resemble interference effects reported in studies on action-perception coupling (Brass et al., 2000, 2001; Kilner et al., 2003). Namely, they may tap incidental and automatic (i.e., “passive”) processes recruited when agents act independently but contingently. Accordingly, it has been suggested that what participants co-represent in these “task sharing” scenarios is *that* another agent is present and *when* it is his/her turn, but not *what* the other agent needs to do (Wenke et al., 2011). On the contrary, shared goals imply that partners have clear in mind both what they need to do (i.e., their own sub-goal), what the partner needs to do (i.e., his/her sub-goal) and their common effects. Hence, shared goals are “active” ingredients of our motor planning: they enable co-agents to dynamically integrate predicted effects of the partner’s action within the agent’s motor plan. In the following section we will explain why a joint-grasping set-up provides an excellent opportunity to investigate the hierarchical structure (where co-agents’ sub-goals depend on overarching shared goals) that—in our view—characterize motor planning during JAs.

## Grasping Kinematics: From Individual Transitive Behavior to Interpersonal Goal Sharing

Prehension, i.e., the capacity to reach and grasp, is the key behavior that allows humans to change their environment, and it has been largely described both in terms of its kinematic features (Jeannerod, 1981, 1984) and in terms of its neural bases (see Castiello, 2005, for a review), thus becoming an “experimental test-case” for the study of transitive, goal directed actions (Grafton, 2010). Indeed, prehension is a somewhat stereotyped movement in which maximum grip aperture (i.e., the thumb-finger maximum distance) is a landmark always occurring at 60–70% of the reach trajectory and highly correlated with object size (Jeannerod, 1981, 1984; see also Bootsma et al., 1994; Smeets and Brenner, 1999). Thus, grasping kinematics follows stereotypical patterns if other factors do not intervene. Importantly, however, grasping kinematics also depends on its desired end-goal. In fact, not only objects features (e.g., texture, weight and fragility; Johansson and Westling, 1988; Weir et al., 1991; Savelsbergh et al., 1996) but also the intentions of an agent (e.g., grasping an object to lift it, to place it in a precise location or to use it, Ansuini et al., 2006, 2008) modify grasping pre-shaping, i.e., the relative position of fingers during the reaching phase, and the contact points of the fingers on the object (Sartori et al., 2011). Finally and most importantly, prehension kinematics is also modulated by social factors as the co-agent’s communicative (Sartori et al., 2009; Ferri et al., 2011; Innocenti et al., 2012) or cooperative/competitive intention (Georgiou et al., 2007; Becchio et al., 2008a,b; see Becchio et al., 2012, for a review). Thus, grasping kinematics becomes an ideal candidate to explore how the socio-emotional context modulates agents’ overt behavior during realistic face-to-face interactions, by using a set-up where object properties (i.e., the physical “sub-goal” of each agent) is kept constant (and cannot thus modulate kinematics) but the co-agents’ “shared” goal and the socio-emotional context are modulated instead. Suggestions have been made that the observation of movement kinematics is what allows an observer to infer the agent’s intention by simply noting details of his/her overt behavior (Ansuini et al., 2014; see also D’Ausilio et al., 2015). For instance, we can distinguish when a given action (say, making a pass) is used for its pragmatic goal (e.g., passing the ball to the teammate) or for a communicative one (e.g., signaling to a co-actor the direction of the pass) from minimal motor cues.

## The Implementation of Shared Goals in a Joint-Grasping Task

**FIGURE 1:**
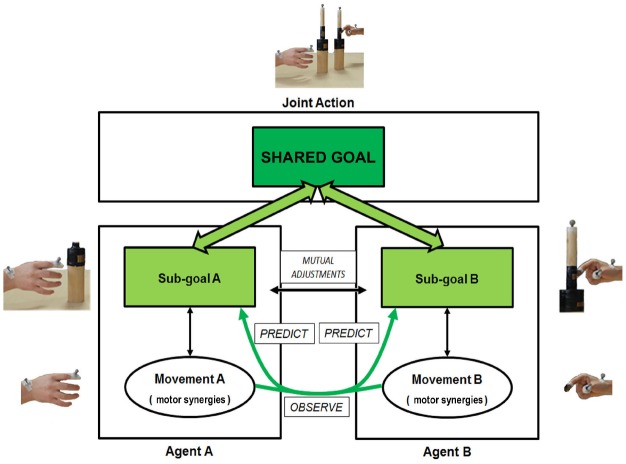
**The figure illustrates how a joint-grasping set-up allows for investigating the hierarchical structure of motor planning which characterizes joint action (JA).** It represents the JA experimental condition described in the main text. Here, each agent’s sub-goal (i.e., grasping the bottle-shaped object at the correct location) depends on the couple’s shared goal (i.e., grasping the object *synchronously* and performing a *complementary/imitative* action, please note in this case a complementary action is shown). Namely, co-agents select the to be grasped object-part according to the shared goal, and perform mutual adjustments in order to fulfill it. Then, the correct movement (i.e., the recruitment of motor synergies to perform a precision/power grip) is selected according to the to be performed sub-goal; however, observing these motor synergies in the partner is a cue in itself, which allows co-agents to predict the partner’s sub-goal and adjust their own movements accordingly.

Taking advantage from early attempts to apply grasping kinematic analysis to research on JA, in recent years we developed a joint-grasping task where each of two individuals sitting one in front of the other is required to reach and grasp a bottle-shaped object. The objects provided to each individual are identical and designed to prompt a precision grip (when grasping a small cylinder in the higher part of the bottle) or a power grip (when grasping a large cylinder in the lower part of the bottle; see Figure 1).

Participants are required to reach-and-grasp the bottle in the correct part following different instructions. Crucially, however, each participant needs to perform the task as synchronously as possible with the partner. The more participants are synchronous, the higher their common payoff. As synchronicity with the partner is essential to fulfill the instructions—in this scenario as well as in many daily life situations—Grasping Asynchrony is the critical dependent variable indexing the success of interpersonal coordination (Sacheli et al., 2012, 2013).

Four features of this paradigm are crucial. Two participants are instructed to perform a face-to-face motor task (i) implying a *shared goal* (i.e., be synchronous) which is dependent on participants’ ability to achieve their own motor sub-goals (i.e., grasping the bottle-shaped object at the correct location), and which also implies that (ii) each participant’s motor sub-goal is dependent on the partner’s action (i.e., the task requires *mutual adjustments*); moreover, in different experimental conditions, participants have (iii) to perform either *imitative* or *complementary* movements with respect to their partner’s one, and (iv) to adjust to the partner’s movements either in *time* only [“synchronization” (Synchr) condition, requiring to be synchronous only] or in time and space [“joint action” (JA) condition, requiring to be synchronous and to adapt to the partner’s sub-goal]. Importantly, in the JA condition participants do not know where to grasp the object in advance: both partners only receive an auditory cue that specifies whether they have to perform an imitative action (precision–precision or power–power grip) or a complementary action (precision-power grip or *vice versa*) as a couple. As a consequence, they have to reciprocally adapt their movements on-line in order to select which action (e.g., precision–precision or power–power grip in case of imitative actions) they are going to perform based on the movement of their partner. Thus, although in principle both Synchr and JA imply a “shared goal” (i.e., be synchronous) according to the Butterfill’s (2012) definition, only the JA condition would capture a situation where participants need to predict the partner’s action and sub-goal in order to select their own action and sub-goal to achieve the shared goal (i.e., not only be synchronous with but also complementary/imitative to your partner): namely, JA requires participants to predict (and represent) *what* the partner is doing (see Figure 1). On the contrary, the partner’s sub-goal might be totally disregarded in the Synchr condition, at least in its spatial features. In this regard, the Synchr condition implies “task sharing” and not necessarily “shared goals” (see the distinction outlined above).

Thanks to such task structure (which includes shared goals) and the peculiar feature of grasping movements, we have been able to explore how co-agents’ (individual) behavior is modulated by socio-emotional variables (Sacheli et al., 2012, 2013, 2015).

**FIGURE 2:**
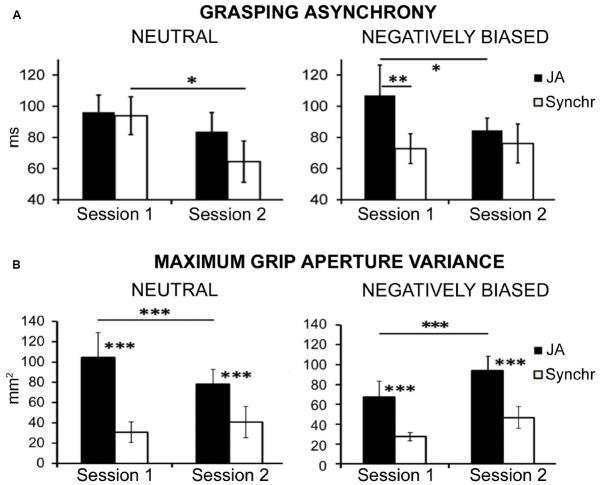
**Modified from Sacheli et al. (2012).** The graphs illustrate results comparing behavioral performance in terms of grasping asynchrony (i.e., time-delay between partners’ grasp times on the bottle, **A**) and grasping kinematics (in terms of maximum grip aperture variance, **B**) of two groups of participants. In one group the interpersonal perception is left neutral (Neutral group), while in the other it is negatively biased (Negatively biased group). **(A)** In the Neutral group, participants initially (session 1) achieve the same level of performance in the synchronization (Synchr) and in the joint action (JA) condition. On the contrary, in the Negatively biased group, participants’ performance in JA is significantly lower (i.e., grasping asynchrony is higher, indicating poorer performance) than in Synchr: this suggests they are acting “each one on his own,” and are thus not able to reciprocally adapt in order to achieve the JA shared goal. **(B)** Coherently, analysis of kinematics reveals that maximum grip aperture is much less variable (indicating less movement corrections and thus less reciprocal adjustments) in the Negatively biased group, supporting the hypothesis that they are less prone to adapt to the partner’s action. However, in the second session maximum grip aperture variance in negatively biased participants increases in JA. This effect is paralleled by an improvement in interpersonal coordination as measured by grasping asynchrony. See the main text for an interpretation of these results. JA, joint action condition; Synchr, synchronization condition. Error bars indicate SEM. *p < 0.05; **p < 0.01; ***p < 0.001.

In one study, by applying this set-up we showed that a negative interpersonal perception (induced by the feeling that the partner has mined one’s own self-esteem, Caprara et al., 1987) strongly modulates the ability to reciprocally coordinate in JA (Sacheli et al., 2012, see Figure 2). Specifically, when participants interact within a negative interpersonal scenario (i.e., negatively biased group), their performance in the JA condition is significantly lower than in the Synchr one (Figure 2A), suggesting they act “each one on their own”: they do not represent the shared goal and hence disregard the partner’s sub-goal, and this impairs the performance when mutual adjustments are required (i.e., in JA). As a matter of fact, the analyses on participants’ movement kinematics demonstrate that participants’ maximum grip aperture in JA is less variable, indicating they perform less movement corrections. This evidence supports the idea that participants are less prone to represent and adapt to the partner’s action and sub-goal (Figure 2B). On the contrary, we showed that in a neutral interpersonal situation pairs of participants achieve the same level of performance in Synchr and JA (Figure 2A): this suggests they represent the task as what Vesper et al. (2010) define a “me + X mode,” i.e., including the partner’s movement in their own motor plan, independently from the experimental condition, namely even when they are not necessarily required to do so (i.e., in the Synchr condition).

Hence, a negative interpersonal bond reduces the tendency to map others’ behavior onto ones’ own sensorimotor system for the purpose of representing the shared goal of the joint-grasping task: participant act independently from each other and do not reciprocally adapt, as if they did not automatically resolve back to a sensorimotor representation of the partner’s movement for the sake of shared goal fulfillment. Conversely, this “shared goal representation” is established in neutral interpersonal situations.

Importantly, in a second session of the experiment, negatively biased participants improve their performance in the JA condition, and this is paralleled by an increase in movement corrections as shown by grasping kinematics. This suggests that acting together might itself facilitate the creation of a social bond between interacting co-agents and change the way partners represent the task: from representing it as an “individual” grasping task where two agents act in synchrony but independently (“task sharing” mode) to representing the task as a joint grasping task having an overarching, cooperative shared goal (“shared goal” mode). Accordingly, one might hypothesize that JA tasks like the one described here might be exploited to investigate whether acting together reduces biases toward other individuals. For instance, we showed that the JA condition is also modulated by racial prejudices (Sacheli et al., 2015), as sensorimotor simulation recruited during JA (indexed by visuo-motor interference measured by the comparison between complementary and imitative actions) negatively correlates with the individual ethnic bias (i.e., it is reduced when interacting with the out-group partner in biased participants only). Studies indicate that unconscious mimicry of others’ postures and mannerisms during interactions may have the social scope of promoting affiliation (Lakin and Chartrand, 2003; van Baaren et al., 2004, 2009), and that the voluntary mimicry of out-group members reduces racial stereotypes (Inzlicht et al., 2012). In a similar vein, the reinforcement of social bonds that arises during prolonged motor interactions (Sacheli et al., 2012) may exert the same powerful modulation.

## Conclusion

The present perspective focuses on the idea that the presence of a shared goal is what qualifies an on-line interaction as being penetrable to interpersonal cues. *Vice versa*, the presence of shared goals during mutually adaptive interactions may promote affiliation between interacting individuals by reinforcing their emotional bond, and this may be reflected in subtle changes in co-agents’ interactional behaviors that can be captured analysing their movement kinematics. The present perspective article intends to suggest that future research on the socio-emotional components of motor interactions do not necessarily require “complex” interactional set-up: indeed, even extremely instrumental and overlearned movements (such as grasping movements) can be shaped by the emotional context in which interaction takes place, provided that the interactive task implies a shared goal.

### Conflict of Interest Statement

The authors declare that the research was conducted in the absence of any commercial or financial relationships that could be construed as a potential conflict of interest.
